# Substance Use and Traumatic Brain Injury: Evidence from a Rural Trauma Center

**DOI:** 10.3390/ijerph23060786

**Published:** 2026-06-11

**Authors:** Monica R. Lininger, Michael Anastario

**Affiliations:** 1 Department of Physical Therapy and Athletic Training, Northern Arizona University, Flagstaff, AZ 86011, USA; 2 Center for Community Health and Engaged Research, Northern Arizona University, Flagstaff, AZ 86011, USA; 3 Department of Health Sciences, Northern Arizona University, Flagstaff, AZ 86011, USA; michael.anastario@nau.edu

**Keywords:** substance use disorder, prevention, traumatic brain injury, rural health, emergency department, health promotion

## Abstract

**Highlights:**

**Public health relevance—How does this work relate to a public health issue?**

- Traumatic brain injury (TBI) and substance use disorder (SUD) frequently co-occur and represent a growing burden in emergency care, particularly in rural populations.
- Rural trauma systems face unique disparities, including higher injury severity, limited access to specialty types of care, and disproportionate impact among American Indian/Alaska Native (AI/AN) patients.

**Public health significance—Why is this work of significance to public health?**

- This study provides novel epidemiological evidence from a large rural dataset (*N* = 24,389 Emergency Department [ED] encounters), addressing a critical gap in understanding the TBI-SUD relationship outside of urban settings.
- Findings highlight demographic disparities, with higher co-diagnosis risk among males and AI/AN patients, and links between alcohol use and injury severity.

**Public health implications—What are the key implications or messages for practitioners, policy makers, and/or researchers in public health?**

- EDs represent a key intervention point for integrated screening and prevention strategies specific to TBI and SUD.
- Rural health systems should implement coordinated, culturally responsive approaches that address the intersection of injury and substance use to reduce morbidity and recurrent healthcare utilization.

**Abstract:**

**Background**: Traumatic brain injury (TBI) and substance use disorder (SUD) frequently co-occur due to shared risk factors and a potentially bidirectional relationship. However, epidemiological patterns in rural populations remain understudied despite known disparities in access and outcomes. This study aimed to characterize the relationship between TBI and SUD in a rural Southwestern population, including demographic and clinical patterns of diagnostic sequencing. **Methods**: A retrospective observational study was conducted using electronic health records and trauma registry data (2022–2023) from a rural trauma center. Cohort one included 24,389 emergency department encounters with ICD-10 codes for TBI or SUD. Cohort two included 248 trauma registry patients with TBI and SUD diagnoses. Descriptive statistics and multinomial logistic regression models were used to evaluate diagnostic patterns and associated demographic factors. **Results**: Males were more likely to have co-occurring TBI and SUD (Relative Risk Ratio [RRR] = 1.35), while increasing age was associated with TBI-only diagnoses. Among patients with multiple visits and diagnoses, 16% had co-diagnoses, while 9% had sequential diagnoses. American Indian/Alaska Native patients had higher co-diagnosis risk compared to White patients (RRR = 2.21, *p* < 0.001). Higher blood alcohol concentration was associated with lower Glasgow Coma Scale scores (r = −0.15, *p* = 0.022), indicating greater severity. **Conclusions**: TBI and SUD frequently co-occur in rural populations, with notable disparities by sex and race/ethnicity. Emergency Departments are critical points of care for interventions such as screening for both substance use and head injury when either is suspected, and employing culturally responsive education and referral pathways upon discharge.

## 1. Introduction

Each year, an estimated 69 million people worldwide sustain a traumatic brain injury (TBI) [1], making it a leading cause of disability and mortality [2,3,4]. Furthermore, it’s postulated that self-reported TBI incidence in adults can range between 21.7% [5] to nearly 50% over the course of a lifetime [6]. In 2013, it was estimated that 2.5 million TBI-related Emergency Department (ED) visits, 282,000 hospitalizations, and 56,000 deaths occurred in the United States (US) [3]. From 2016–2018, there were 181,227 TBI-related deaths, with higher rates in states with a large proportion of residents living in rural areas [7]. These estimates are similar to those from 2020–2021 [8], highlighting that this public health concern is not going away. Additionally, Shaik et al. analyzed TBI-related death rates using surveillance data from the Centers for Disease Control and Prevention from 1999–2020 and found that the TBI-related age-adjusted mortality rate was stable during this time period. Beyond the burden of the prevalence of TBIs, there are also long-term cognitive impairments [9], psychiatric comorbidity [10], and elevated risk for neurodegenerative diseases [6,11].

Another equal public health concern is substance use in the US. In 2023, 48.5 million people in the US aged 12 or older (17.1%) met criteria for a past-year substance use disorder (SUD) [12]. In 2024, there were 79,384 drug overdose deaths in the US, with an age-adjusted rate of 23.1 deaths per 100,000 [13]. Since the onset of the COVID-19 pandemic, both the prevalence of substance use disorders and the health complications linked to them have risen [14]. Substance use contributes substantially to acute care utilization, with national emergency department surveillance data showing high volumes of polysubstance use involvement and alcohol-related emergency department visits [15]. In the US, EDs are being considered critical venues for engaging people who use drugs and for implementing interventions for SUD [16]. Given that both TBI and SUD frequently result in emergency care utilization, understanding how these conditions overlap and interact in these patients has become an increasingly important area of public health interest.

The interaction between substance use and head trauma is complex, perhaps bidirectional, and not clearly understood [17,18]. Some have documented that substance use is a risk factor for TBI [19,20,21]. Others have demonstrated an increase in substance use after a TBI, potentially attributable to mood disorders and neurobehavioral changes [18,22]. Although substantial evidence supports a relationship between TBI and SUD, some studies have reported inconsistent findings on whether TBI independently increases the risk for later substance misuse when accounting for demographic, psychiatric, and behavioral confounders [23,24]. Mechanistically, substance use may increase TBI risk through impaired judgment, slower reaction time, and engagement in high-risk behaviors [18,25]. Conversely, TBI may increase a person’s vulnerability to increased substance use based on challenges in impulse control [26], emotional regulation [27], and post-injury psychiatric conditions such as depression [10,28] and anxiety [10,21]. Most recently, Hoglund et al. used data from the TriNetX Research Network, which includes over 1.8 million patients with TBI, and found that the incidence of new SUD over the following 5 years was 4.2% [29].

While evidence is growing in this area regarding the connection between TBI and SUD, most of it is drawn from national datasets or large urban hospital systems, leaving knowledge about more rural settings limited. Because rural trauma systems are often the primary point of care for both acute head injuries and substance-use-related emergencies, these settings might provide important insights into how TBI and SUD intersect with the medically underserved population. Gabella et al. found that severity and mortality were higher in rural communities than in urban areas in Colorado [30]. This aligns directly with the work of Brown et al., which used geospatial analysis to identify higher fatality rates in nonmetropolitan counties [31]. Severity and mortality are not the only measured outcomes; successful discharge rates [32] and functional status [33] have also been found to be significantly lower for patients with TBI in rural settings compared to those in urban communities. To address gaps and inconsistencies in knowledge regarding the epidemiology and demographic correlates of the connection between SUD and TBI, especially in rural communities, this exploratory retrospective study characterized diagnostic patterns, demographic associations, and clinical severity indicators (e.g., Glasgow Coma Scale [GCS], blood alcohol concentration [BAC], trauma team activation) in a cohort of patients from a trauma center in the Southwestern United States. We hypothesized that co-occurring TBI and SUD diagnoses would be observed in at least 10% of this rural population and that demographic characteristics such as age, sex, and race/ethnicity would be statistically associated with diagnostic patterns and clinical severity indicators.

## 2. Materials and Methods

### 2.1. Participant and Data Source

In this retrospective observational study, data were extracted from an electronic health record (EHR) of a trauma center in the Southwestern United States. The study was conducted in accordance with the Strengthening the Reporting of Observational Studies in Epidemiology guidelines [34] and is reported in Supplementary S1. Ethical approval was not required for this study as it used a de-identified retrospective dataset, in accordance with the Human Research Protection Program policy regarding non-human subjects research. The data analyzed did not contain any HIPAA-defined identifiers or Protected Health Information. The hospital system also approved the ethical practices of this study through its internal research review board. Data included two patient cohorts from the 2022–2023 period. Records and ICD-10 codes were requested for: all substance use-related codes (F10.1 through F19.9); overdose/poisoning codes (T40.0–T40.9 and T51.0–T51.9); and concussion-related codes (S01.0–S09.9 and T90.1, T90.2, T90.4, T90.5, T90.8, and T90.9). All ICD-10 parent codes with diagnostic groupings are presented in Supplementary S2.

The first cohort comprised individuals (*N* = 24,389) seen in the ED who were not admitted, and the trauma team was not activated, with ICD-10 diagnosis codes for SUD or TBI. Within cohort 1, among the subset of patients who had both multiple visits and multiple diagnosis codes, we constructed a nominal outcome representing diagnostic pattern: TBI diagnosis documented prior to SUD diagnosis, SUD diagnosis documented prior to TBI diagnosis, co-diagnosis of TBI and SUD within the same visit, TBI diagnosis only, and SUD diagnosis only (reference category).

The second cohort (*n* = 248) was directly from the trauma registry for the hospital, with individuals seen through the ED in cases where the trauma team was activated for those with a TBI and then an SUD diagnosis. The second cohort was different from the first, as only individuals with a TBI who also had subsequent ICD-10 codes associated with substance use were included. Additionally, those patients in the second cohort required more critical care (i.e., trauma team activation) than those in the first cohort. A patient could be included in both cohorts, but not for the same visit, because diagnosis codes for each encounter could be included only in the general EHR (Cohort 1) or the trauma registry (Cohort 2).

### 2.2. Statistical Analysis

The analysis strategy was designed to characterize and evaluate SUD and TBI diagnoses using patient data. Since data from Cohort one and Cohort two were substantially different, separate analytic plans were used for each to accommodate differences in sample size and variables. Analyses were primarily exploratory and descriptive in nature and were intended to characterize patterns within the available clinical dataset rather than establish causal relationships. All statistical analyses were conducted in STATA 18.0 and Spyder 5.0 (Python 3.12.4; packaged by Anaconda, Inc., Austin, TX, USA).

For Cohort one (*N* = 24,389), patient encounters were classified into four visit types based on the number of visits and number of diagnosis codes: one visit with one diagnosis code, one visit with multiple diagnosis codes, multiple visits with one diagnosis code, and multiple visits with multiple diagnosis codes. Differences in patient age across visit types were evaluated using one-way Analysis of Variance (ANOVA). Associations between visit type and patient gender and race/ethnicity were assessed using the Pearson chi-square test. Multinomial logistic regression models were used to estimate associations between diagnostic patterns and patient age, gender, and race/ethnicity, which were treated as independent variables. Outcome specificity for age was evaluated using Wald chi-square tests of equality of coefficients across multinomial logit equations to assess whether age effects differed across diagnostic pattern categories. Gender effects were evaluated by examining outcome-specific multinomial coefficients. Given sparse cell counts for certain race/ethnicity cells, outcome specificity was evaluated using a joint Wald chi-square test of race coefficients across multinomial outcome equations.

For Cohort two (*n* = 248), descriptive statistics were used to characterize patient demographics, clinical presentation, and substance exposure. Patient age, GCS score, and BAC were summarized using means and standard deviations. Gender, race/ethnicity, transport mode, primary payor, and toxicology results were summarized using frequencies and percentages, including instances of missingness.

Bivariate associations between race/ethnicity and toxic substances detected on admission were evaluated using Pearson chi-square tests. Differences in mean blood alcohol concentration across race/ethnicity categories were assessed using one-way ANOVA. Differences in mean GCS score across race/ethnicity categories were also evaluated using one-way ANOVA. The association between blood alcohol concentration and neurologic status was examined using Pearson correlation coefficients. To further evaluate predictors of blood alcohol concentration, a multivariable linear regression model was estimated with blood alcohol concentration as the outcome and GCS score, race/ethnicity, and age as independent variables. Race/ethnicity was included as a categorical predictor. Model coefficients were interpreted as absolute differences in blood alcohol concentration.

Missing data were handled using complete-case (available-case) analysis, with observations excluded only for variables missing within a given analysis where applicable. Multicollinearity was assessed using variance inflation factors (VIFs), and no VIF exceeded 2.0, indicating no evidence of problematic multicollinearity among predictors. Assumptions for the multivariable linear regression model were evaluated through inspection of residual plots, normal probability plots, tests for heteroskedasticity, and influence diagnostics. Supplemental analyses using heteroskedasticity-robust standard errors were also conducted to evaluate model stability. Sparse race/ethnicity categories in multinomial logistic regression were evaluated descriptively and are acknowledged as a limitation due to unstable estimates associated with small cell counts. Given the exploratory nature of the analyses, formal adjustment for multiple comparisons was not performed.

## 3. Results

Both cohorts were predominantly male, with males comprising 58.2% of Cohort one (14,199 of 24,386) and 79.8% of Cohort two (198 of 248), indicating a substantially higher male representation in Cohort two. American Indian/Alaska Native (AI/AN) individuals represented a substantially larger proportion of Cohort two (48.8%, 121 of 248) compared to Cohort one (20.3%, 4867 of 24,011), while White individuals constituted a smaller proportion of Cohort two (36.3%, 90 of 248) relative to Cohort one (65.2%, 15,651 of 24,011).

### 3.1. Cohort One Findings

A total of 24,389 patient records were included in the analyses concerning visit type. Patient age differed significantly across visit types (F(3, 24,382) = 82.07, *p* < 0.001), with patients with multiple diagnosis codes generally younger (>1 visit with one diagnosis code: 53.8 ± 18.3; >1 visit with multiple diagnosis codes: 48.9 ± 18.6) than those with a single diagnosis code (single visit with one diagnosis code: 46.2 ± 22.9; single visit with multiple diagnosis codes: 44.4 ± 20.4). Visit type was also associated with patient gender (χ^2^(3) = 218.17, *p* < 0.001), with males being overrepresented in categories containing multiple diagnostic codes (Table 1). Visit type was also significantly associated with race/ethnicity (χ^2^(21) = 496.66, *p* < 0.001), with AI/AN patients overrepresented in categories with multiple diagnostic codes (Table 1). The most common diagnosis was SUD (*n* = 8098), with most cases within the single visit with multiple diagnoses group (2066, 63.4%). Head injury was second (*n* = 7345), following the same trend of most patients seen with multiple diagnoses in a single visit (1675, 51.4%). It was uncommon for someone with SUD or a TBI to be seen multiple times, with only this single diagnosis (SUD: 132 patients, 9.3%; Head injury: 80 patients, 5.6%).

Visit type was strongly associated with diagnostic content. The prevalence of head injury diagnoses differed across visit types (χ^2^(3) ≈ 1400, *p* < 0.001), as did the prevalence of SUD diagnoses (χ^2^(3) ≈ 2800, *p* < 0.001) and poisoning diagnoses (χ^2^(3) = 148.20, *p* < 0.001). Head injury diagnoses were most prevalent (31.5%) for patients with a single visit containing one diagnostic code, whereas SUD diagnoses and poisoning diagnoses were most prevalent for single visits with multiple diagnostic codes (63.4% and 5.7%, respectively; Table 1).

**Figure 1:**
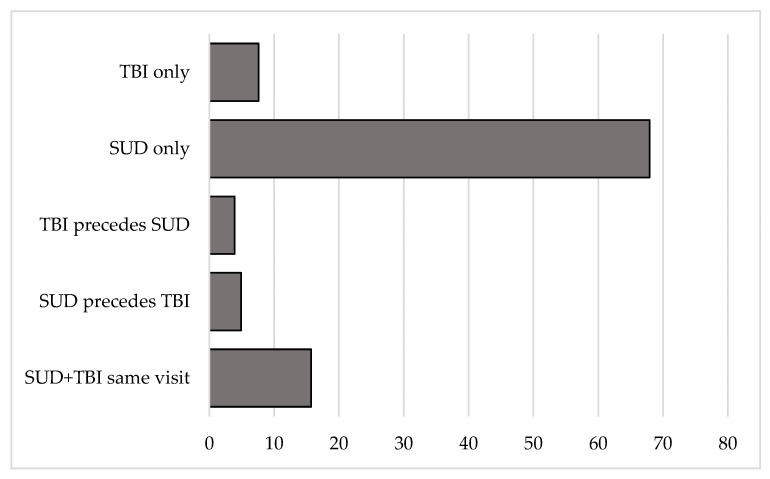
Percentages of SUD and TBI diagnoses codes for those patients with more than a single visit and multiple diagnosis codes (*n* = 4790) in Cohort one (TBI only = 7.6%, SUD only = 67.9%, TBI precedes TBI = 3.9%, SUD precedes TBI = 4.9%, SUD and TBI in the same visit = 15.7%). Acronyms: TBI = traumatic brain injury, SUD = substance use disorder.

In the subsample of Cohort one patients with multiple visits and multiple diagnosis codes (*n* = 4768), 67.9% had SUD-only diagnoses, 15.7% were co-diagnosed with TBI and SUD within the same visit, 7.6% had TBI-only diagnoses, 4.9% had SUD diagnoses documented prior to TBI diagnoses, and 3.9% had TBI diagnoses documented prior to SUD diagnoses (Figure 1). Multinomial logistic regression models were estimated using SUD-only as the reference outcome and White patients as the reference race category (Table 2). Age showed outcome-specific associations with diagnostic sequence (Wald χ^2^(3) = 71.54, *p* < 0.001). Increasing age was associated with a higher relative risk of TBI-only diagnoses (RRR = 1.03 per year; 95% CI: 1.02–1.04; *p* < 0.001) and of SUD diagnoses documented prior to TBI (RRR = 1.02 per year; 95% CI: 1.01–1.02; *p* < 0.001) but was not significantly associated with co-diagnosis or with TBI diagnoses documented prior to SUD. Compared with females, males had a higher relative risk of co-diagnosis (RRR = 1.35; *p* < 0.01) and a lower relative risk of TBI-only diagnoses (RRR = 0.60; *p* < 0.001), relative to SUD-only diagnoses; sex was not significantly associated with the remaining diagnostic sequence categories. Race/ethnicity was jointly associated with diagnostic sequence (Wald χ^2^(28) = 124.02, *p* < 0.001), indicating heterogeneous race/ethnicity associations across outcome categories. Compared with white patients, AI/AN patients had a higher relative risk of co-diagnosis (RRR = 2.21; *p* < 0.001) (Table 2).

### 3.2. Cohort Two Findings

Cohort two consisted of 248 patients with a mean age of 39.1 years (SD = 16.2) (Table 3). The mean Glasgow Coma Scale (GCS) score was 11.7 (SD = 4.8), and the mean blood alcohol concentration was 0.11% (SD = 0.13). The cohort was predominantly male (79.8%). Nearly half of patients identified as AI/AN (48.8%), followed by White patients (36.3%); other racial groups each comprised small proportions of the cohort. Most patients arrived by ambulance, with helicopter transport (45.2%) and ground ambulance transport (44.4%) accounting for the majority of presentations, while 7.7% arrived as walk-ins. Medicaid or State insurance was the most common primary payor (60.1%), followed by private commercial insurance (14.9%) and Medicare (9.3%), with smaller proportions covered by other government programs or self-pay (Table 3).

The distribution of toxic substances detected differed significantly by race/ethnicity in Cohort two (Pearson χ^2^(36) = 87.10, *p* < 0.001; Table 4a). AI/AN patients accounted for the largest proportion of positive toxicology findings across most substance categories. Tetrahydrocannabinol (THC)/marijuana was the most frequently detected substance among AI/AN patients (56.2%), followed by stimulants (22.3%), with smaller proportions testing positive for benzodiazepines (5.8%), opiates (5.0%), and barbiturates (3.3%). Patterns among White patients were similar for THC/marijuana but showed a lower prevalence of stimulant detection and a higher proportion of untested cases (Table 4a).

Mean blood alcohol concentration differed significantly by race/ethnicity (F(6, 231) = 5.92, *p* < 0.001; Table 4b). AI/AN patients had the highest mean blood alcohol concentration (0.16%, SD = 0.14). Mean GCS scores among AI/AN patients were similar to those of White patients and other racial groups, indicating comparable neurologic status at presentation (Table 4b). Blood alcohol concentration was weakly but significantly negatively correlated with GCS score (r = −0.15, *p* = 0.022), indicating lower GCS scores with increasing alcohol levels.

In a multivariable linear regression model predicting blood alcohol concentration, higher alcohol levels were independently associated with lower GCS scores (β = −0.004 per one-point increase in GCS, *p* = 0.030), after adjustment for race/ethnicity and age. Relative to the reference race category (not documented), AI/AN patients had significantly higher blood alcohol concentrations (β = 0.08, 95% CI: 0.02 to 0.13; *p* = 0.004). No other race/ethnicity categories were significantly associated with blood alcohol concentration, although estimates for several groups were imprecise due to small sample sizes. Age was not significantly associated with blood alcohol concentration (*p* = 0.090) (Table 5).

## 4. Discussion

This work aimed to characterize and examine the relationship between SUD and TBI in a Southwestern rural population, using two distinct yet complementary data sources to enhance the generalizability of the results. More specifically, diagnostic care patterns, the influence of demographic variables, and clinical severity indicators were assessed. Main findings included that there was a strong overlap between TBI and SUD in this rural population, that sex and age shaped diagnostic patterns, and AI/AN patients were overrepresented in both cohorts. These findings should be interpreted as exploratory observational associations derived from retrospective clinical data.

Among patients with multiple visits and diagnoses (cohort 1), nearly 16% had a co-diagnosis in the same encounter, consistent with our hypothesis that both conditions should be considered during ED visits. Although these findings should not be interpreted as evidence of causal directionality, they reinforce the importance of considering both simultaneously during a medical evaluation. This pattern is consistent with prior literature, suggesting that acute substance exposure may contribute to circumstances associated with head injury through impaired judgment, reaction time, and balance, therefore increasing the risk of falls, motor vehicle accidents, and interpersonal violence [17,25]. Co-occurrence of SUD and TBI for individuals with TBI admitted to a trauma hospital setting has been reported as high as 47% in an Eastern Norway trauma hospital [25]. Others have found that between 23–51% of adolescents and adults who have suffered a TBI were intoxicated at the time of the occurrence [35,36]. These estimates could be high, as the diagnosis was substance use, not SUD.

Demographic factors, such as sex and age, were significantly associated with diagnostic patterns. Males were more likely than females to experience a co-diagnosis of TBI and SUD and less likely to have only a TBI diagnosis. It is well documented that males engage in more risk behavior than females. Recent evidence [37] has also shown that males are more likely to be intoxicated at the time of a TBI, aligning with the present findings. However, there is conflicting evidence on the role that sex plays in TBI when evaluating rates alone. According to the CDC, males are almost two times more likely to be hospitalized for a TBI, compared to females [8]. Conversely, in sport-related head trauma, females experience a higher risk compared to males, even in the same sport with similar rules [38,39,40]. An increase in age was also associated with a higher risk of a TBI-only diagnosis as well as an SUD diagnosis documented prior to a TBI diagnosis. Individuals over 75 have the highest rates and account for over 30% of all TBI-related hospitalizations [8]. The most common mechanism in this age group is falls [41,42]. The substance use before TBI could also be a culmination of substance use exposure over the course of one’s lifetime. Interestingly, age was not significantly associated with TBI documented prior to SUD, which contradicts some recent findings that TBI could be a risk factor for subsequent alcohol abuse [29]. However, some work with those in the military found that the presence of a TBI was not associated with increased levels of alcohol use [43,44]. The influence of sex and age on diagnostic patterns suggests that individuals seeking care at EDs with TBI and SUD may not present uniformly across patient groups.

Race was also an influential factor in the present study. AI/AN have the highest average annual TBI-related deaths compared to any other ethnic group [45,46,47]. However, despite this growing concern, research in this population remains limited [48]. Currently, there are two postulates explaining why rates among AI/AN are so high. First, violent mechanisms, like intimate partner violence, are seen in AI/AN women at five times the national average [49,50]. Second, it may be substance use, specifically alcohol, which is nearly twice that of individuals in other ethnic groups [51]. Data from the Indian Health Service (IHS) National Patient Information Reporting System from 2005–2014 indicated that higher rates of TBI-related ED visits may be due to increased SUD in this population [52]. This research team also reported that the age-adjusted rate of ED visits for TBI was highest among AI/AN patients living in the Southwest compared with other IHS regions [52], which aligns with the location of the current work. While research on TBI and SUD has been challenging or severely limited in AI/AN populations, preliminary evidence suggests that these outcomes may overlap and synergistically contribute to excess disease burden.

Although this work presents novel findings in a rural population, there are limitations. Small sample sizes in some race categories reinforce the need for a more focused AI/AN analysis, rather than a broad analysis of racial categories, as was done here. As with all retrospective studies using medical records, there is a risk for incomplete or missing data, which may under-identify SUD or misclassify TBI diagnoses. Given the exploratory nature of several subgroup and association analyses, the findings should be interpreted with caution, particularly in light of multiple comparisons and limited sample sizes. The blood alcohol concentration outcome showed mild heteroskedasticity and slight deviation from normality, although supplemental analyses using heteroskedasticity-robust standard errors yielded similar conclusions. Additionally, because the study relied on secondary retrospective clinical data, the timing and procedures for the GCS assessment and BAC measurement methods could not be standardized or verified, potentially leading to measurement error and testing bias. Furthermore, this study was based on retrospectively available clinical data, and no prospective sample size or power calculations were performed. The results are also limited to a single Southwestern rural trauma center, which significantly limits the generalizability of the findings. Finally, the study included only a 2-year time frame, which may limit long-term understanding of TBI or SUD after the prior diagnosis. Future research should prioritize long-term follow-up, which would allow for stronger claims on one diagnosis being documented prior to another.

## 5. Conclusions

Together, these findings suggest a strong and complex intersection between TBI and SUD in a rural Southwestern population, with meaningful heterogeneity by sex, age, and race/ethnicity. The high frequency of co-diagnosis and the proportion of patients receiving both diagnoses within the same encounter support emergency department-based strategies that address overdose, intoxication, and head injury as linked clinical problems. The overrepresentation of AI/AN patients in both cohorts, coupled with higher alcohol concentrations at presentation, points to a need for interventions that are culturally tailored and responsive. These results also highlight the need for SUD screening in TBI examination and post-treatment [29]. Rural trauma systems may be one of the few consistent points of contact for patients at the intersection of injury and substance-related harm, making them a pragmatic locale for interventions that seek to reduce recurrent emergency department use and prevent morbidity and mortality. Specifically, screening protocols could be integrated for both intoxication and head injuries when either is suspected. Additionally, clear referral-to-treatment pathways could be established locally, accompanied by culturally responsive discharge education.

## Figures and Tables

**Table 1:** Distribution of Visit Type by Demographic Characteristic for Patients (*N* = 24,389) in Cohort One. Frequencies (percentages) of patients are stratified by single-visit versus multiple-visit status and by one diagnosis code versus multiple diagnosis codes, by gender, and by race/ethnicity.

Characteristic	Single Visit, One Diagnosis Code	Single Visit, Multiple Diagnoses Codes	>1 Visit, One Diagnosis Code	>1 Visit, Multiple Diagnoses Codes
	Frequency (percent)			
Total Patients	14,916 (61.2)	3261 (13.4)	1422 (5.8)	4790 (19.6)
Gender ***				
Females	6622 (44.4)	1097 (33.6)	708 (49.8)	1760 (36.7)
Males	8292 (55.6)	2163 (66.3)	714 (50.2)	3030 (63.3)
Undifferentiated	2 (0.01)	1 (0.03)	0 (0)	0 (0)
Total	14,916	3261	1422	4790
Race/Ethnicity ***				
American Indian/Alaska Native	2554 (17.5)	920 (28.7)	153 (10.8)	1240 (26.0)
Asian	141 (1.0)	26 (0.8)	3 (0.2)	9 (0.2)
Black/African American	248 (1.7)	43 (1.3)	24 (1.7)	60 (1.3)
Hispanic	1466 (10.0)	285 (8.9)	122 (8.6)	331 (6.9)
Mixed Race	191 (1.3)	24 (0.7)	9 (0.6)	33 (0.7)
Multiple Races	233 (1.6)	68 (2.1)	19 (1.3)	106 (2.2)
Hawaiian/Other Pacific Islander	41 (0.30)	3 (0.09)	3 (0.2)	5 (0.1)
White	9751 (66.7)	1832 (57.2)	1084 (76.5)	2984 (62.6)
Total	14,625	3201	1417	4768

*** *p* < 0.001.

**Table 2:** Multinomial Logistic Regression of Diagnostic Sequencing Patterns with Relative Risk Ratios (95% CIs) for Patients in Cohort 1.

Predictor	Co-Diagnosed vs. SUD Only	SUD Precedes TBI vs. SUD Only	TBI Only vs. SUD Only	TBI Precedes SUD vs. SUD Only
Age (years)	1.00 (0.99, 1.00)	1.02 *** (1.01, 1.02)	1.03 *** (1.02, 1.04)	1.00 (0.99, 1.01)
Male	1.35 ** (1.13, 1.61)	0.92 (0.70, 1.21)	0.60 *** (0.48, 0.75)	1.03 (0.75, 1.40)
Race/Ethnicity				
American Indian/Alaska Native	2.21 *** (1.84, 2.64)	0.99 (0.70, 1.41)	0.95 (0.69, 1.30)	0.84 (0.57, 1.24)
Black/African American	0.87 (0.39, 1.95)	0.96 (0.30, 3.15)	0.23 (0.03, 1.70)	1.56 (0.55, 4.41)
Hispanic	1.02 (0.72, 1.46)	1.44 (0.89, 2.34)	1.52 * (1.01, 2.27)	1.17 (0.67, 2.05)
Mixed Race	0.99 (0.34, 2.90)	—	4.19 ** (1.63, 10.77)	0.77 (0.10, 5.83)
Multiple Races	2.40 *** (1.50, 3.84)	1.07 (0.38, 3.00)	1.06 (0.42, 2.68)	1.91 (0.85, 4.30)
Constant	0.15 *** (0.11, 0.20)	0.03 *** (0.02, 0.05)	0.03 *** (0.02, 0.05)	0.06 *** (0.04, 0.10)

Acronyms: CIs = confidence intervals; SUD = substance use disorder; TBI = traumatic brain injury. Reference outcome category: Substance Use Disorder only. Reference categories for predictors: Female (sex), White (race). Race categories Asian and Native Hawaiian or Other Pacific Islander were excluded from the table due to sparse cells and quasi-complete separation, resulting in unstable estimates. * *p* < 0.05, ** *p* < 0.01, *** *p* < 0.001.

**Table 3:** Demographic, Clinical, and Transport Characteristics of Patients in Cohort 2.

Characteristic	Mean ± Standard Deviation
Age (years) (*n* = 248)	39.09 ± 16.21
GCS score (*n* = 246)	11.70 ± 4.80
Blood alcohol %, (*n* = 238)	0.11 ± 0.13
	**Frequency (Percent)**
Gender (*n* = 248)	
Female	50 (20.16)
Male	198 (79.84)
Missing	0 (0)
Race/Ethnicity (*n* = 248)	
American Indian/Alaska Native	121 (48.79)
Asian	2 (0.81)
Black/African American	5 (2.02)
Hawaiian/Pacific Islander	1 (0.40)
Other	1 (0.40)
White	90 (36.29)
Missing	28 (11.29)
Transport mode (*n* = 248)	
Fixed-wing ambulance	3 (1.21)
Ground ambulance	110 (44.35)
Helicopter ambulance	112 (45.16)
Police	1 (0.40)
POV/Walk-in	19 (7.66)
Missing	3 (1.21)
Primary payor (*n* = 248)	
Medicaid	149 (60.08)
Medicare	23 (9.27)
Other	2 (0.81)
Other government	13 (5.24)
Private-commercial	37 (14.92)
Self pay	22 (8.87)
Workers comp	1 (0.40)
Missing	1 (0.40)

Acronyms: POV = privately owned vehicle.

**Table 4:** (**a**) Toxic Substances Detected by Race/Ethnicity for Patients in Cohort 2 with Frequency (Percent) (*n* = 248). (**b**) Alcohol Concentration and Neurologic Status by Race/Ethnicity for Patients in Cohort 2.

(**a**)								
**Race**	**Stimulant**	**Barbiturate**	**Benzodiazepine**	**None**	**Not Tested**	**Opiates**	**THC/Marijuana**	**Total**
American Indian/ Alaska Native	27 (22.31)	4 (3.31)	7 (5.79)	4 (3.31)	5 (4.13)	6 (4.96)	68 (56.20)	121
Other *	10 (27.03)	1 (2.70)	1 (2.70)	1 (2.70)	2 (5.41)	1 (2.70)	21 (56.76)	37
White	18 (20.00)	0 (0.00)	3 (3.33)	2 (2.22)	9 (10.00)	8 (8.89)	50 (55.56)	90
Total	55 (22.18)	5 (2.02)	11 (4.44)	7 (2.82)	16 (6.45)	15 (6.05)	139 (56.05)	248
(**b**)								
	**Blood Alcohol Percent**				**Glasgow Coma Scale Score**			
**Race**	**Frequency**		**Mean ± Standard Deviation**		**Frequency**		**Mean ± Standard Deviation**	
American Indian/Alaska Native	117		0.16 ± 0.14		119		11.54 ± 4.83	
White	85		0.07 ± 0.10		90		12.03 ± 4.73	
Black/African American	5		0.04 ± 0.08		5		11.40 ± 5.37	
Asian	2		0.00 ± 0.00		2		11.00 ± 5.66	
Hawaiian/Pacific Islander	1		0.27 ± —		1		3.00 ± —	
Other	1		0.00 ± —		1		14.00 ± —	
Not documented	27		0.09 ± 0.13		28		11.64 ± 4.89	

Acronyms: THC = Tetrahydrocannabinol. * Race and ethnicity categories with sparse cell counts were collapsed for presentation clarity. Full disaggregated results are available in Supplementary S3 ANOVA (blood alcohol × race): F(6, 231) = 5.92, *p* < 0.001; ANOVA (GCS × race): F(6, 239) = 0.69, *p* = 0.6.

**Table 5:** Multivariable Linear Regression Predicting Blood Alcohol Concentration for Patients in Cohort Two (*n* = 236) *.

Predictor	β Coefficient	Standard Error	*p*-Value	95% CI
GCS score	−0.004	0.002	0.030	−0.01, −0.00
American Indian/Alaska Native	0.08	0.03	0.004	0.02, 0.13
Asian	−0.09	0.09	0.323	−0.27, 0.09
Black/African American	−0.05	0.06	0.402	−0.17, 0.07
Hawaiian/Pacific Islander	0.14	0.13	0.254	−0.10, 0.39
Other	−0.08	0.13	0.542	−0.32, 0.17
White	−0.01	0.03	0.615	−0.07, 0.04
Age (years)	−0.00	0	0.090	−0.00, 0.00
Constant	0.16	0.04	<0.001	0.09, 0.23

Acronyms: GCS = Glasgow Coma Scale; CIs = confidence intervals. Reference category of not documented race. * Supplemental analyses using heteroskedasticity-robust standard errors yielded substantively similar conclusions.

## Data Availability

The data associated with this manuscript will not be shared due to the privacy and confidentiality of medical records.

## Supplementary Materials

The following supporting information can be downloaded at https://www.mdpi.com/article/10.3390/ijerph23060786/s1: Table S1: STROBE Statement—Checklist of items that should be included in reports of cohort studies [34]; Table S2: Substance Use Disorder ICD-10 Codes, Diagnostic Description, and Clinical Category; Table S3: Head Injury and TBI-Related ICD-10 Codes, Diagnostic Description, and Clinical Category; Table S4: Overdose and Poisoning ICD-10 Codes, Diagnostic Description, and Clinical Category; Table S5: Toxic Substances Detected by Race for Patients in Cohort 2 with Frequency (Percent) (*n* = 248).

## Abbreviations

The following abbreviations are used in this manuscript:

AI/AN	American Indian/Alaska Native
ANOVA	Analysis of Variance
BAC	Blood Alcohol Concentration
CI	Confidence Interval
ED	Emergency Department
EHR	Electronic Health Record
GCS	Glasgow Coma Scale
ICD-10	International Classification of Diseases, 10th Revision
POV	Privately Owned Vehicle
RRR	Relative Risk Ratio
SD	Standard Deviation
SUD	Substance Use Disorder
TBI	Traumatic Brain Injury
US	United States

## References

[1] Dewan M.C., Rattani A., Gupta S., Baticulon R.E., Hung Y.-C., Punchak M., Agrawal A., Adeleye A.O., Shrime M.G., Rubiano A.M. (2018). Estimating the Global Incidence of Traumatic Brain Injury. J. Neurosurg..

[2] Chen Z., Wang Z., Mentis A.-F.A., Stey A.M., Schwulst S.J. (2024). Factors Associated with Unfavorable Outcomes in Older Patients with Traumatic Brain Injury: Analysis from the “All of Us” Research Program. Front. Neurol..

[3] Taylor C.A. (2017). Traumatic Brain Injury–Related Emergency Department Visits, Hospitalizations, and Deaths—United States, 2007 and 2013. Morb. Mortal. Wkly. Rep. Surveill. Summ..

[4] Johnson W.D., Griswold D.P. (2017). Traumatic Brain Injury: A Global Challenge. Lancet Neurol..

[5] Corrigan J.D., Yang J., Singichetti B., Manchester K., Bogner J. (2018). Lifetime Prevalence of Traumatic Brain Injury with Loss of Consciousness. Inj. Prev. J. Int. Soc. Child Adolesc. Inj. Prev..

[6] Maas A.I.R., Menon D.K., Adelson P.D., Andelic N., Bell M.J., Belli A., Bragge P., Brazinova A., Büki A., Chesnut R.M. (2017). Traumatic Brain Injury: Integrated Approaches to Improve Prevention, Clinical Care, and Research. Lancet Neurol..

[7] Daugherty J., Hong Z., Sarmiento K., Waltzman D. (2021). Differences in State Traumatic Brain Injury–Related Deaths, by Principal Mechanism of Injury, Intent, and Percentage of Population Living in Rural Areas—United States, 2016–2018. MMWR Morb. Mortal. Wkly. Rep..

[8] Centers for Disease Control and Prevention TBI Data. https://www.cdc.gov/traumatic-brain-injury/data-research/index.html.

[9] Schneider A.L.C., Pike J.R., Elser H., Coresh J., Mosley T.H., Diaz-Arrastia R., Gottesman R.F. (2024). Traumatic Brain Injury and Cognitive Change over 30 Years among Community-Dwelling Older Adults. Alzheimers Dement. J. Alzheimers Assoc..

[10] Gould K.R., Ponsford J.L., Johnston L., Schönberger M. (2011). The Nature, Frequency and Course of Psychiatric Disorders in the First Year after Traumatic Brain Injury: A Prospective Study. Psychol. Med..

[11] Fann J.R., Ribe A.R., Pedersen H.S., Fenger-Grøn M., Christensen J., Benros M.E., Vestergaard M. (2018). Long-Term Risk of Dementia among People with Traumatic Brain Injury in Denmark: A Population-Based Observational Cohort Study. Lancet Psychiatry.

[12] SAMHSA (2023). Highlights for the 2023 National Survey on Drug Use and Health. https://www.samhsa.gov/data/sites/default/files/NSDUH%202023%20Annual%20Release/2023-nsduh-main-highlights.pdf.

[13] Garnett M.F., Miniño A.M. (2026). Drug overdose deaths in the United States, 2023–2024. NCHS Data Brief..

[14] Venkatesh A.K., Janke A.T., Kinsman J., Rothenberg C., Goyal P., Malicki C., D’Onofrio G., Taylor A., Hawk K. (2022). Emergency Department Utilization for Substance Use Disorders and Mental Health Conditions during COVID-19. PLoS ONE.

[15] SAMHSA (2024). Drug Abuse Warning Network (DAWN): Alcohol-Related ED Visits Short Report. https://library.samhsa.gov/product/dawn-alcohol-related-short-report/pep24-07-019.

[16] Reardon A.W.T., Kim H.S. (2026). The Importance of Addiction Medicine Education for Emergency Medicine Residents and Opportunities to Increase Uptake of Evidence-Based Interventions for Substance Use Disorder. Ann. Emerg. Med..

[17] Olsen C.M., Corrigan J.D. (2022). Does Traumatic Brain Injury Cause Risky Substance Use or Substance Use Disorder?. Biol. Psychiatry.

[18] Olson-Madden J.H., Brenner L.A., Corrigan J.D., Emrick C.D., Britton P.C. (2012). Substance Use and Mild Traumatic Brain Injury Risk Reduction and Prevention: A Novel Model for Treatment. Rehabil. Res. Pract..

[19] Bombardier C.H., Rimmele C.T., Zintel H. (2002). The Magnitude and Correlates of Alcohol and Drug Use before Traumatic Brain Injury. Arch. Phys. Med. Rehabil..

[20] Rogers J.M., Read C.A. (2007). Psychiatric Comorbidity Following Traumatic Brain Injury. Brain Inj..

[21] Ponsford J., Alway Y., Gould K.R. (2018). Epidemiology and Natural History of Psychiatric Disorders After TBI. J. Neuropsychiatry Clin. Neurosci..

[22] Newman S.D., Grantz J.G., Brooks K., Gutierrez A., Kawata K. (2020). Association between History of Concussion and Substance Use Is Mediated by Mood Disorders. J. Neurotrauma.

[23] Graham D.P., Cardon A.L. (2008). An Update on Substance Use and Treatment Following Traumatic Brain Injury. Ann. N. Y. Acad. Sci..

[24] Bjork J.M., Grant S.J. (2009). Does Traumatic Brain Injury Increase Risk for Substance Abuse?. J. Neurotrauma.

[25] Andelic N., Jerstad T., Sigurdardottir S., Schanke A.-K., Sandvik L., Roe C. (2010). Effects of Acute Substance Use and Pre-Injury Substance Abuse on Traumatic Brain Injury Severity in Adults Admitted to a Trauma Centre. J. Trauma Manag. Outcomes.

[26] Vonder Haar C., Martens K.M., Riparip L.-K., Rosi S., Wellington C.L., Winstanley C.A. (2017). Frontal Traumatic Brain Injury Increases Impulsive Decision Making in Rats: A Potential Role for the Inflammatory Cytokine Interleukin-12. J. Neurotrauma.

[27] Merkel S.F., Cannella L.A., Razmpour R., Lutton E., Raghupathi R., Rawls S.M., Ramirez S.H. (2017). Factors Affecting Increased Risk for Substance Use Disorders Following Traumatic Brain Injury: What We Can Learn from Animal Models. Neurosci. Biobehav. Rev..

[28] Singh R.K., Humphries T.J., Dawson J.F., Tiupin-Szulc J., Mason S., Lecky F.E. (2025). Changes in Depression Symptoms over 10 Years after TBI; a Long-Term Prospective Study. Brain Inj..

[29] Hoglund Z.T., Sollenberger C., Scott K.W., Arena J.D., Srinivasan V.M., Burkhardt J.-K., Turnbull J., Rosado-Philippi J., Heitkotter H., Helfand A.I. (2026). Relative Incidence of New-Onset Substance Use Disorders Following Traumatic Brain Injury: A Global Retrospective Multicenter Analysis Using the TriNetX Database. J. Clin. Med..

[30] Gabella B., Hoffman R.E., Marine W.W., Stallones L. (1997). Urban and Rural Traumatic Brain Injuries in Colorado. Ann. Epidemiol..

[31] Brown J.B., Kheng M., Carney N.A., Rubiano A.M., Puyana J.C. (2019). Geographical Disparity and Traumatic Brain Injury in America: Rural Areas Suffer Poorer Outcomes. J. Neurosci. Rural Pract..

[32] Anderson M.C., Evans E., Zonfrillo M.R., Thomas K.S. (2021). Rural/Urban Differences in Discharge from Rehabilitation in Older Adults with Traumatic Brain Injury. J. Am. Geriatr. Soc..

[33] Schootman M., Fuortes L. (1999). Functional Status Following Traumatic Brain Injuries: Population-Based Rural-Urban Differences. Brain Inj..

[34] von Elm E., Altman D.G., Egger M., Pocock S.J., Gøtzsche P.C., Vandenbroucke J.P. (2007). STROBE Initiative Strengthening the Reporting of Observational Studies in Epidemiology (STROBE) Statement: Guidelines for Reporting Observational Studies. BMJ.

[35] Cuthbert J.P., Harrison-Felix C., Corrigan J.D., Kreider S., Bell J.M., Coronado V.G., Whiteneck G.G. (2015). Epidemiology of Adults Receiving Acute Inpatient Rehabilitation for a Primary Diagnosis of Traumatic Brain Injury in the United States. J. Head Trauma Rehabil..

[36] Parry-Jones B.L., Vaughan F.L., Miles Cox W. (2006). Traumatic Brain Injury and Substance Misuse: A Systematic Review of Prevalence and Outcomes Research (1994-2004). Neuropsychol. Rehabil..

[37] Oliverio R., Karelina K., Weil Z.M. (2020). Sex, Drugs, and TBI: The Role of Sex in Substance Abuse Related to Traumatic Brain Injuries. Front. Neurol..

[38] Centers for Disease Control and Prevention Data on Sports and Recreation Activities. https://www.cdc.gov/heads-up/data/index.html.

[39] Covassin T., Moran R., Elbin R.J. (2016). Sex Differences in Reported Concussion Injury Rates and Time Loss From Participation: An Update of the National Collegiate Athletic Association Injury Surveillance Program From 2004–2005 Through 2008–2009. J. Athl. Train..

[40] Kerr Z.Y., Chandran A., Nedimyer A.K., Arakkal A., Pierpoint L.A., Zuckerman S.L. (2019). Concussion Incidence and Trends in 20 High School Sports. Pediatrics.

[41] Thompson H.J., McCormick W.C., Kagan S.H. (2006). Traumatic Brain Injury in Older Adults: Epidemiology, Outcomes, and Future Implications. J. Am. Geriatr. Soc..

[42] Moreland B., Kakara R., Henry A. (2020). Trends in Nonfatal Falls and Fall-Related Injuries Among Adults Aged ≥65 Years—United States, 2012-2018. MMWR Morb. Mortal. Wkly. Rep..

[43] Miles S.R., Graham D.P., Teng E.J. (2015). Examining the Influence of Mild Traumatic Brain Injury and Posttraumatic Stress Disorder on Alcohol Use Disorder in OEF/OIF Veterans. Mil. Med..

[44] Heltemes K.J., Dougherty A.L., MacGregor A.J., Galarneau M.R. (2011). Alcohol Abuse Disorders among U.S. Service Members with Mild Traumatic Brain Injury. Mil. Med..

[45] Centers for Disease Control and Prevention (2022). Surveillance Report of Traumatic Brain Injury-Related Deaths by Age Group, Sex, and Mechanism of Injury—United States, 2018 and 2019.

[46] Peterson A., Thomas K., Kegler S. (2025). Disparities in Traumatic Brain Injury-Related Deaths-the United States, 2021. Brain Inj..

[47] Maldonado J., Huang J.H., Childs E.W., Tharakan B. (2023). Racial/Ethnic Differences in Traumatic Brain Injury: Pathophysiology, Outcomes, and Future Directions. J. Neurotrauma.

[48] Watson J.D., Perrin P.B., Arango-Lasprilla J.C. (2025). Disparities between Native Americans and White Individuals in Global Outcome Trajectories over the 5 Years after Traumatic Brain Injury: A Model Systems Study. PLoS ONE.

[49] St. Ivany A., Schminkey D. (2016). Intimate Partner Violence and Traumatic Brain Injury: State of the Science and Next Steps. Fam. Community Health.

[50] Malcoe L.H., Duran B.M., Montgomery J.M. (2004). Socioeconomic Disparities in Intimate Partner Violence against Native American Women: A Cross-Sectional Study. BMC Med..

[51] Linton K.F., Kim B.J. (2016). The Moderation of Blood Alcohol Levels on Higher Odds of Survival among American Indians with Violent, Blunt-Force Traumatic Brain Injuries. Soc. Work Public Health.

[52] Sarmiento K., Kennedy J., Daugherty J., Peterson A.B., Evans M.E., Haberling D.L., Billie H. (2020). Traumatic Brain Injury-Related Emergency Department Visits Among American Indian and Alaska Native Persons-National Patient Information Reporting System, 2005-2014. J. Head. Trauma Rehabil..

