# Supplementary material for: Substance Use and Traumatic Brain Injury: Evidence from a Rural Trauma Center
Source: Int J Environ Res Public Health. 2026 Jun 11;23(6):786. doi: 10.3390/ijerph23060786 (PMC13300210; doi:10.3390/ijerph23060786)
Supplement: Supplementary file 1 [file ijerph-23-00786-s001.zip › Supplementary S1.pdf]

Supplementary S1

Supplementary S1.1

Table S1. STROBE Statement—Checklist of items that should be included in reports of cohort studies [34].

	Item No	Recommendation	Page, Section
Title and abstract	1	(a) Indicate the study's design with a commonly used term in the title or the abstract	1, Abstract Methods
		(b) Provide in the abstract an informative and balanced summary of what was done and what was found	1-2, Abstract
Introduction			
Background/ rationale	2	Explain the scientific background and rationale for the investigation being reported	2, Introduction paragraphs 1-3
Objectives	3	State specific objectives, including any prespecified hypotheses	3, Introduction, paragraph 4
Methods			
Study design	4	Present key elements of study design early in the paper	3, 2.1 Participant and Data Source
Setting	5	Describe the setting, locations, and relevant dates, including periods of recruitment, exposure, follow-up, and data collection	3-4, 2.1 Participant and Data Source
Participants	6	(a) Give the eligibility criteria, and the sources and methods of selection of participants. Describe methods of follow-up	4, 2.1 Participant and Data Source
		(b) For matched studies, give matching criteria and number of exposed and unexposed	n/a
Variables	7	Clearly define all outcomes, exposures, predictors, potential confounders, and effect modifiers. Give diagnostic criteria, if applicable	4, 2.2 Statistical Analysis
Data sources/ measurement	8*	For each variable of interest, give sources of data and details of methods of assessment (measurement). Describe comparability of assessment methods if there is more than one group	4, 2.2 Statistical Analysis
Bias	9	Describe any efforts to address potential sources of bias	4, 2.1 Participant and Data Source; 8, Table 2; Appendix C
Study size	10	Explain how the study size was arrived at	4, 2.2 Statistical Analysis
Quantitative variables	11	Explain how quantitative variables were handled in the analyses. If applicable, describe which groupings were chosen and why	4, 2.2 Statistical Analysis
Statistical methods	12	(a) Describe all statistical methods, including those used to control for confounding	4, 2.2 Statistical Analysis
		(b) Describe any methods used to examine subgroups and interactions	4, 2.2 Statistical Analysis

		(c) Explain how missing data were addressed	4, 2.2 Statistical Analysis
		(d) If applicable, explain how loss to follow-up was addressed	n/a
		(e) Describe any sensitivity analyses	n/a
Results			
Participants	13*	(a) Report numbers of individuals at each stage of study—eg numbers potentially eligible, examined for eligibility, confirmed eligible, included in the study, completing follow-up, and analysed	5, 3.1 Cohort One Findings; 6, Table; 9, 3.2 Cohort Two Findings; 10, Table 3
		(b) Give reasons for non-participation at each stage	n/a
		(c) Consider use of a flow diagram	n/a
Descriptive data	14*	(a) Give characteristics of study participants (eg demographic, clinical, social) and information on exposures and potential confounders	5, 3.1 Cohort One Findings; 6, Table; 9, 3.2 Cohort Two Findings; 10, Table 3
		(b) Indicate number of participants with missing data for each variable of interest	10, Table 3
		(c) Summarise follow-up time (eg, average and total amount)	n/a
Outcome data	15*	Report numbers of outcome events or summary measures over time	n/a
Main results	16	(a) Give unadjusted estimates and, if applicable, confounder-adjusted estimates and their precision (eg, 95% confidence interval). Make clear which confounders were adjusted for and why they were included	9, 3.2. Cohort two findings
		(b) Report category boundaries when continuous variables were categorized	6, Figure; 7, Table 1
		(c) If relevant, consider translating estimates of relative risk into absolute risk for a meaningful time period	5, 3.1 Cohort One Findings
Other analyses	17	Report other analyses done—eg analyses of subgroups and interactions, and sensitivity analyses	11, Table 4b; Appendix C
Discussion			
Key results	18	Summarise key results with reference to study objectives	13, Discussion paragraph 1
Limitations	19	Discuss limitations of the study, taking into account sources of potential bias or imprecision. Discuss both direction and magnitude of any potential bias	14,
Interpretation	20	Give a cautious overall interpretation of results considering objectives, limitations, multiplicity of analyses, results from similar studies, and other relevant evidence	15, Conclusions
Generalisabil-ity	21	Discuss the generalisability (external validity) of the study results	15, Conclusions
Other information			

Funding	22	Give the source of funding and the role of the funders for the present study and, if applicable, for the original study on which the present article is based	16, Funding
