# Supplementary material for: Substance Use and Traumatic Brain Injury: Evidence from a Rural Trauma Center
Source: Int J Environ Res Public Health. 2026 Jun 11;23(6):786. doi: 10.3390/ijerph23060786 (PMC13300210; doi:10.3390/ijerph23060786)
Supplement: Supplementary file 1 [file ijerph-23-00786-s001.zip › Supplementary S2.pdf]

SupplementaryS2

Supplementary S2.1

Table S2. Substance Use Disorder ICD-10 Codes, Diagnostic Description, and Clinical Category.

ICD-10 Code	Diagnosis Description	Clinical Category
F10.1	Alcohol use	
F10.2	Alcohol dependence	
F10.3	Alcohol withdrawal	
F10.4	Alcohol withdrawal delirium	
F10.5	Alcohol-induced psychotic disorder	Alcohol related disorders
F10.6	Alcohol-induced amnestic disorder	
F10.7	Alcohol-induced residual and late-onset psychotic disorder	
F10.8	Other alcohol-induced disorders	
F10.9	Alcohol use, unspecified	
F11.1	Opioid use	
F11.2	Opioid dependence	
F11.3	Opioid withdrawal	
F11.4	Opioid-induced delirium	
F11.5	Opioid-induced psychotic disorder	Opioid related disorders
F11.6	Opioid-induced persisting dementia	
F11.7	Opioid-induced persisting amnestic disorder	
F11.8	Other opioid-induced disorders	
F11.9	Opioid use, unspecified	
F12.1	Cannabis use	
F12.2	Cannabis dependence	
F12.3	Cannabis withdrawal state	
F12.4	Cannabis withdrawal state with delirium	
F12.5	Cannabis-induced psychotic disorder	Cannabis related disorders
F12.6	Cannabis-induced persisting dementia	
F12.7	Cannabis-induced persisting amnestic disorder	
F12.8	Other cannabis-induced disorders	
F12.9	Cannabis use, unspecified	

F13.1	Sedative, hypnotic, or anxiolytic-related use	Sedative, hypnotic, or anxiolytic related disorders
F13.2	Sedative, hypnotic, or anxiolytic-related dependence	
F13.3	Sedative, hypnotic, or anxiolytic-related withdrawal	
F13.4	Sedative, hypnotic, or anxiolytic-related withdrawal delirium	
F13.5	Sedative, hypnotic, or anxiolytic-related psychotic disorder	
F13.6	Sedative, hypnotic, or anxiolytic-related amnestic disorder	
F13.7	Sedative, hypnotic, or anxiolytic-related residual and late-onset psychotic disorder	
F13.8	Other sedative, hypnotic, or anxiolytic-related mental and behavioral disorders	
F13.9	Sedative, hypnotic, or anxiolytic-related use, unspecified	
F14.1	Cocaine use	Cocaine related disorders
F14.2	Cocaine dependence	
F14.3	Cocaine withdrawal	
F14.4	Cocaine-induced intoxication delirium	
F14.5	Cocaine-induced psychotic disorder	
F14.6	Cocaine-induced amnestic disorder	
F14.7	Cocaine-induced residual/late-onset disorder	
F14.8	Other cocaine-induced disorders	
F14.9	Cocaine use, unspecified	
F15.1	Other stimulant use	Other stimulant related disorders
F15.2	Other stimulant dependence	
F15.3	Other stimulant withdrawal state	
F15.4	Other stimulant withdrawal state with delirium	
F15.5	Other stimulant-induced psychotic disorder	
F15.6	Other stimulant-induced amnestic syndrome	
F15.7	Other stimulant-induced residual and late-onset psychotic disorder	
F15.8	Other stimulant-induced disorders	
F15.9	Unspecified stimulant use, unspecified	
F16.1	Hallucinogen use	Hallucinogen related disorders
F16.2	Hallucinogen dependence	
F16.3	Hallucinogen withdrawal	
F16.4	Hallucinogen-induced intoxication delirium	
F16.5	Hallucinogen-induced psychotic disorder	
F16.6	Hallucinogen-induced amnestic disorder	
F16.7	Hallucinogen-induced residual and late-onset psychotic disorder	

F16.8	Other mental and behavioral disorders due to hallucinogen use	
F16.9	Hallucinogen use, unspecified	
F18.1	Inhalant use	Inhalant related disorders
F18.2	Inhalant dependence	
F18.3	Inhalant withdrawal	
F18.4	Inhalant-induced delirium	
F18.5	Inhalant-induced psychotic disorder	
F18.6	Inhalant-induced persisting amnestic disorder	
F18.7	Inhalant-induced residual and late-onset psychotic disorder	
F18.8	Inhalant-induced other mental and behavioral disorders	
F18.9	Inhalant use, unspecified	
F19.1	Other psychoactive substance use	Other psychoactive substance related disorders
F19.2	Other psychoactive substance dependence	
F19.3	Other psychoactive substance withdrawal	
F19.4	Other psychoactive substance-induced delirium	
F19.5	Other psychoactive substance-induced psychotic disorder	
F19.6	Other psychoactive-induced persisting amnestic disorder	
F19.7	Other psychoactive residual and late-onset psychotic disorder	
F19.8	Other psychoactive mental and behavioral disorders	
F19.9	Other psychoactive substance use, unspecified	

Supplementary S2.2

Table S3. Head Injury and TBI-Related ICD-10 Codes, Diagnostic Description, and Clinical Category.

ICD-10 Code	Diagnosis Description	Clinical Category
S01.0	Open wound of scalp	Open wounds of the head
S01.1	Open wound of eyelid and periocular area	
S01.2	Open wound of nose	
S01.3	Open wound of ear	
S01.4	Open wound of cheek and temporomandibular area	
S01.5	Open wound of lip and oral cavity	
S01.6	Open wound of larynx and trachea	
S01.7	Multiple open wounds of head	
S01.8	Open wound of other parts of head	
S01.9	Open wound of unspecified part of head	
S02.0	Fracture of vault of skull	Fracture of skull and facial bones
S02.1	Fracture of base of skull	
S02.2	Fracture of nasal bones	
S02.7	Multiple fractures involving skull and facial bones	
S02.8	Fracture of other specified skull and facial bones	
S02.9	Fracture of skull and facial bones, unspecified	
S04.0	Injury of optic nerve and pathways	Injury of optic nerve and pathways
S06.0	Concussion	Intracranial injury
S06.1	Traumatic cerebral edema	
S06.2	Diffuse traumatic brain injury	
S06.3	Focal traumatic brain injury	
S06.4	Epidural hemorrhage	
S06.5	Traumatic subdural hemorrhage	
S06.6	Traumatic subarachnoid hemorrhage	
S06.7	Intracranial injury with loss of consciousness	
S06.8	Other specified intracranial injuries	
S06.9	Unspecified intracranial injury	
S07.0	Crushing injury of face	Crushing injuries to the head
S07.1	Crushing injury of skull	

S07.8	Crushing injury of other parts of head	
S07.9	Crushing injury of head, part unspecified	
S09.7	Multiple injuries of head	Other and unspecified injuries of head
S09.8	Other specified injuries of head	
S09.9	Unspecified injury of face and head	
T90.1	Sequelae of open wound of head	Sequelae of injuries of head
T90.2	Sequelae of fracture of skull and facial bones	
T90.4	Sequelae of injury of eye and orbit	
T90.5	Sequelae of intracranial injury	
T90.8	Sequelae of other specified injuries of head	
T90.9	Sequelae of unspecified injury of head	

Table S4. Overdose and Poisoning ICD-10 Codes, Diagnostic Description, and Clinical Category.

ICD-10 Code	Diagnosis Description	Clinical Category
T40.0	Poisoning by, adverse effect of, or underdosing of opium	Poisoning by, adverse effect of, and underdosing of narcotics and psychodysleptics
T40.1	Poisoning by, adverse effect of, or underdosing of heroin	
T40.2	Poisoning by, adverse effect of, or underdosing of natural and semi-synthetic opioids	
T40.3	Poisoning by, adverse effect of, or underdosing of methadone	
T40.4	Poisoning by, adverse effect of, or underdosing of synthetic narcotics	
T40.5	Poisoning by, adverse effect of, or underdosing of cocaine	
T40.6	Poisoning by, adverse effect of, or underdosing of other and unspecified narcotics	
T40.7	Poisoning by, adverse effect of, or underdosing of cannabis (derivatives)	
T40.8	Poisoning by, adverse effect of, or underdosing of lysergide	
T40.9	Poisoning by, adverse effect of, or underdosing of other and unspecified psychodysleptics	
T51.0	Toxic effect of ethanol	Toxic effect of alcohol
T51.1	Toxic effect of methanol	
T51.4	Toxic effect of toxic drinks	
T51.8	Toxic effect of other specified alcohols	
T51.9	Toxic effect of unspecified alcohol	
