# Supplementary material for: Substance Use and Traumatic Brain Injury: Evidence from a Rural Trauma Center
Source: Int J Environ Res Public Health. 2026 Jun 11;23(6):786. doi: 10.3390/ijerph23060786 (PMC13300210; doi:10.3390/ijerph23060786)
Supplement: Supplementary file 1 [file ijerph-23-00786-s001.zip › Supplementary S3.pdf]

Supplementary S3

Supplementary S3.1

Table S5. Toxic Substances Detected by Race for Patients in Cohort 2 with Frequency (Percent) (N = 248).

Race	Stimulant	Barbiturate	Benzodiazepine	None	Not tested	Opiates	THC/marijuana	Total
Not documented	8 (28.57)	0 (0.00)	1 (3.57)	0 (0.00)	2 (7.14)	0 (0.00)	17 (60.71)	28
American Indian/ Alaska Native	27 (22.31)	4 (3.31)	7 (5.79)	4 (3.31)	5 (4.13)	6 (4.96)	68 (56.20)	121
Asian	0 (0.00)	0 (0.00)	0 (0.00)	1 (50.00)	0 (0.00)	1 (50.00)	0 (0.00)	2
Black/African American	2 (40.00)	0 (0.00)	0 (0.00)	0 (0.00)	0 (0.00)	0 (0.00)	3 (60.00)	5
Hawaiian /Pacific Islander	0 (0.00)	1 (100.00)	0 (0.00)	0 (0.00)	0 (0.00)	0 (0.00)	0 (0.00)	1
Other	0 (0.00)	0 (0.00)	0 (0.00)	0 (0.00)	0 (0.00)	0 (0.00)	1 (100.00)	1
White	18 (20.00)	0 (0.00)	3 (3.33)	2 (2.22)	9 (10.00)	8 (8.89)	50 (55.56)	90
Total	55 (22.18)	5 (2.02)	11 (4.44)	7 (2.82)	16 (6.45)	15 (6.05)	139 (56.05)	248

Acronyms: THC = Tetrahydrocannabinol

Pearson chi-square: $\chi^2(36) = 87.10$, $p < 0.001$.
